# Hybrid PWM Techniques for a DCM-232 Three-Phase Transformerless Inverter with Reduced Leakage Ground Current

**DOI:** 10.3390/mi13010036

**Published:** 2021-12-28

**Authors:** Gerardo Vazquez-Guzman, Panfilo R. Martinez-Rodriguez, Jose M. Sosa-Zuñiga, Dalyndha Aztatzi-Pluma, Diego Langarica-Cordoba, Belem Saldivar, Rigoberto Martínez-Méndez

**Affiliations:** 1 Laboratory of Electrical and Power Electronics, Tecnologico Nacional de Mexico/ITS de Irapuato, Irapuato 36821, GTO, Mexico; gerardo.vazquez@ieee.org (G.V.-G.); jmsosa@ieee.org (J.M.S.-Z.); 2 School of Sciences, Universidad Autonoma de San Luis Potosi (UASLP), San Luis Potosi 78295, SLP, Mexico; diego.langarica@uaslp.mx; 3 Department of Mechatronics Engineering, Tecnologico Nacional de Mexico/ITS de Abasolo, Abasolo 36976, GTO, Mexico; dalyndha.ap@abasolo.tecnm.mx; 4 Faculty of Engineering, Autonomous University of the State of Mexico, Toluca 50130, MEX, Mexico; mbsaldivarma@conacyt.mx (B.S.); rmartinezme@uaemex.mx (R.M.-M.); 5 Cátedras CONACYT, Ciudad de Mexico 03940, CDMX, Mexico

**Keywords:** PV generation, space vector modulation, transformerless inverters, grid connection, leakage ground current

## Abstract

Pulse Width Modulation (PWM) strategies are crucial for controlling DC–AC power converters. In particular, transformerless inverters require specific PWM techniques to improve efficiency and to deal with leakage ground current issues. In this paper, three hybrid PWM methods are proposed for a DCM-232 three-phase topology. These methods are based on the concepts of carrier-based PWM and space vector modulation. Calculations of time intervals for active and null vectors are performed in a conventional way, and the resulting waveforms are compared with a carrier signal. The digital signals obtained are processed using Boolean functions, generating ten signals to control the DCM-232 three-phase inverter. The performance of the three proposed PWM methods is evaluated considering the reduction in leakage ground current and efficiency. The proposed modulation techniques have relevant performances complying with international standards, which make them suitable for transformerless three-phase photovoltaic (PV) inverter markets. To validate the proposed hybrid PWM strategies, numerical simulations and experimental tests were performed.

## 1. Introduction

Transformerless photovoltaic (PV) systems have increased their popularity due to their high performance in terms of efficiency, size, and price. Nevertheless, the loss of galvanic isolation involves other challenges, for instance, reducing or eliminating leakage currents (LKC) that appear in the ground path.

In three-phase transformerless PV systems, conventional topologies such as the six-switch three-phase inverter or the three-phase cascade multilevel inverter (3P-CMI) generate high-frequency common-mode voltage (CMV) components, which due to the structure of the system, cause the common-mode current (CMC), also known as LKC. The CMC is the major issue in transformerless grid connected PV systems, as it can lead to additional power losses, protection tripping, important safety problems, and high total harmonic distortion (THD) of the current injected into the grid. Due to these issues, international norms have been developed to limit the CMC circulation through the PV system to guarantee the security of the system and humans in contact with it, for instance, the international standard DIN VDE 0126-1-1 establishes the maximum limit of the RMS value of the CMC in 300 mA. The scientific community has reported several techniques based on different approaches to mitigate the CMC. Among these, pulse width modulation (PWM) solutions have been popular because it is not necessary to increase the number of semiconductors in the topology or to implement complex control systems. Furthermore, additional functions such as voltage balancing of capacitors can be performed in split DC bus topologies as in neutral point clamped (NPC) inverters, and total system efficiency can be improved through a proper modulation design.

The six-switch three-phase inverter (3P-FB) has been widely studied under space vector modulation techniques for transformerless grid connection and motor drive applications. In [1], a near-state PWM (NSPWM) method was proposed; here, a comparison with similar PWM methods is performed. The NSPWM method makes use of a set of three voltage vectors to match the output and volt-second references. The three voltage vectors are two adjacent vectors together with a near-neighbor vector; then, nonzero voltage vectors are utilized. The sectors are displaced from each other by 30${}^{\circ }$; therefore, new regions are defined with respect to the conventional distribution. As no zero voltage vectors are used, the CMV does not take values produced by those vectors; therefore, CMV variations are reduced. In the case of PV systems, in [2], an evaluation of three-phase converters without galvanic isolation is reported. The analysis considers the conventional 3P-FB, the 3P-FB with split capacitor (3P-FBSC), and the three-phase NPC inverter (3P-NPC). The results demonstrate that the 3P-FBSC and 3P-NPC inverters produce low CMC, which make them two suitable solutions in three-phase transformerless inverters.

Derived from the 3P-FB inverter topology, some modified topologies have been also proposed to solve the CMC issue. For example, by adding passive components, as in the Z-source inverter topology presented in [3], it is possible to avoid the use of a boost stage at the input of the system. Additionally, the CMC magnitude can be reduced by modifying the PWM strategy as in [4], where the authors proposed a modified Z-source inverter and a space vector based modulation (SVM) technique that reduces the CMC magnitude. Moreover, in [5,6,7], a family of topologies called Quasi Z-source inverters is presented, the main idea is to reduce the component rating, the source stress, and component count and to make some contributions to simplify the control strategies. Another approach consists in the addition of active components in order to deal with the CMC, for instance, diodes, IGBTs, or MOSFETs as in [8], where additional diodes connected as a three-phase rectifier plus an IGBT are used to implement the null vectors in an SVM technique. The main idea is to connect the output of the inverter to Vdc/3 or to 2Vdc/3, thus avoiding some CMV transitions and reducing the magnitude of the CMC. Another alternative based on the traditional 3P-FB inverter is to use the idea of DC decoupling developed in the H5 and H6 topologies for single-phase systems [9,10]. In this case, [11,12] proposed the H7 topology and a study of several SVM techniques to reduce the CMC, and [13] proposed that the H8 topology and its SVM technique are presented. In both cases, the main idea is to disconnect the DC bus during the null vectors, which in combination with an adequate sequence of active vectors allows us to mitigate the CMC. Another topology that is a combination of a NPC topology and the 3P-FB inverters is presented in [14] where a type of NPC circuit is added at the output of the inverter following the idea of the HERIC single-phase inverter [15]. The null vectors are now implemented in a freewheeling circuit, which reduces the transitions of the CMV in the circuit, obtaining a reduction in the CMC magnitude.

The DCM-232 topology and its space vector PWM strategy have been designed to deal with the CMC issue. The main objective as in the aforementioned cases is to reduce the fast changes in the CMV by as much as possible and consequently to decrease the magnitude of the CMC. This inverter is based also on the 3P-FB topology at the AC output side, while on the DC input side, there are two DC sources that can be completely decoupled from the 3P-FB circuit by means of two semiconductors switched at the same time [16]. The PWM strategy is based on the space vector PWM technique, where the main difference is that the active vectors are implemented in the 3P-FB circuit and that the zero vectors are implemented by decoupling the DC sources using power semiconductor switches. In the literature, some PWM strategies have also been designed for the DCM-232 inverter; see for instance [17], where a carrier-based PWM is proposed to solve the CMC issue using the principle explained above.

In this paper, three PWM methods based on the Space Vector PWM (SVPWM) technique are studied to reduce the CMC components. The proposed modulation strategies are used to control the three-phase DCM-232 topology. The time intervals to control on and off conditions for each switch are defined using the waveforms obtained by means of the SVPWM concept and then a comparison with a triangular carrier signal is performed. Finally, the resulting signals are processed by a Boolean function implemented in a Complex Programable Logic Device (CPLD) to determine the final sequence for each switch. The DCM-232 topology consists in a 3P-FB inverter plus four switches that decouples the signal to generate two independent DC sources, i.e., two PV generators. The main idea is to control the decoupling switches in order to keep the CMV constant, thereby achieving a reduction in the CMC. The CMV evaluation is performed by driving the Common Mode Model (CMM) of the DCM-232 inverter. In addition, the paper considers an efficiency analysis based on numerical results obtained by means of the implementation of the real models of the semiconductors. Finally, a comparative analysis between the proposed PWM techniques and some solutions available in the literature is performed.

## 2. Topology Description and Proposed Space Vector PWM Techniques

**Figure 1:**
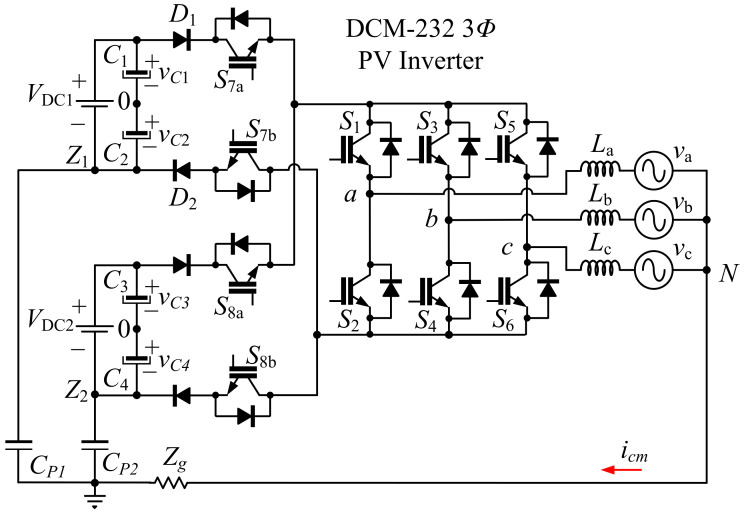
DCM-232 three-phase transformerless PV inverter topology.

A simplified circuit of the DCM-232 topology considered for the design of the SVPWM techniques is shown in Figure 1. One of the main considerations for the simplified circuit is that the DC sources are assumed constant. However, in a real PV transformerless system, the voltage magnitude is slightly variable and, in that case, it is necessary to implement a solution, for example, modifying the modulation strategy or implementing a balance control loop; nevertheless, this topic is out of the scope of this paper and is left for a future research. Three additional important considerations of the simplified diagram are that only the stray capacitance generated by the PV panel are considered; the drive circuits for the semiconductors and control system are not included because these elements do not affect the common mode behavior. Finally, in the case of the ground impedance, the capacitive and inductive effects are disregarded in the system. Therefore, the impedance is considered mainly resistive [18]. The different states that can be proposed to control the DCM-232 inverter in which the structure consists of ten switches are summarized in Table 1. As it can be observed, there are eight possible states. These states produce the following voltage levels between phases and the neutral connection: $V_{DC1}$ = $V_{DC2}$, $2V_{DC}/3$, $V_{DC}/3$, 0V, $-V_{DC}/3$, and $-2V_{DC}/3$. It is important to note that all of the states considered here are exactly the same as in the conventional 3P-FB inverter. However, the main difference is that the switches on the DC side, $S_{7a}$,$S_{7b}$, $S_{8a}$, and $S_{8b}$, are used to make a decoupling action when certain active or null vectors supply the load.

### 2.1. DCM-232 Common Mode Model

**Figure 2:**
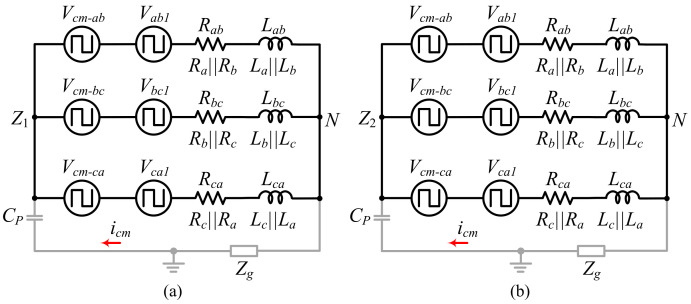
DCM-232 common mode model: (**a**) $V_{DC1}$ and (**b**) $V_{DC2}$.

In order to determine the CMV behavior, a CMM for the DCM-232 topology is derived. Considering the directions given in [19], the simplified CMM shown in Figure 2 can be obtained. As it can be observed, there are two separated circuits, the circuit in Figure 2a corresponds to the DC source $V_{DC1}$, while the simplified circuit shown in Figure 2b corresponds to the DC source $V_{DC2}$. Since the power sources are isolated from each other with a common load, the obtained model is also separated and is essentially the model obtained in [2] for the 3P-FB inverter. Based on that, the common mode voltage in this topology can be calculated in each DC source using (1) and (2). Considering (1) and (2), the CMV can be calculated for each state defined in Table 1, and the results are shown in Table 2. It can be noted that the CMV maintains the same magnitude throughout the switching period when the odd vectors ($V_{1}$, $V_{3}$ and $V_{5}$) or the even vectors ($V_{2}$, $V_{4}$ and $V_{6}$) are connected to the load using $V_{DC1}$ and $V_{DC2}$, respectively. On the other hand, when zero vectors are generated ($V_{0}$ and $V_{7}$), the CMV keeps the previous value because both DC sources are decoupled from the load.

(1) $$V_{CMV1}=\frac{V_{aZ1}+V_{bZ1}+V_{cZ1}}{3},$$

(2) $$V_{CMV2}=\frac{V_{aZ2}+V_{bZ2}+V_{cZ2}}{3}.$$

### 2.2. Proposed Space Vector PWM Techniques

The circuit theory states that a three-phase system can be represented on a α-β plane by means of the Clarke transformation, as shown in (3)–(5). Note that a simplification using cosine functions for the three-phase voltage components ($v_{a}$, $v_{b}$, and $v_{c}$) is considered.

(3) $$\left[\begin{array}{c} v_{\alpha }\\ v_{\beta } \end{array}\right]=\frac{2}{3}\left[\begin{array}{ccc} 1 & -\frac{1}{2} & -\frac{1}{2}\\ 0 & \frac{\sqrt{3}}{2} & -\frac{\sqrt{3}}{2} \end{array}\right]\left[\begin{array}{c} v_{a}\\ v_{b}\\ v_{c} \end{array}\right],$$

(4) $$v_{\alpha }=V_{m}\cos\left(\omega t\right),$$

(5) $$v_{\beta }=V_{m}\sin\left(\omega t\right).$$

Based on (4) and (5), the module and the angle of the reference vector, $V_{ref}$, can be calculated as

(6) $$|V_{ref}|=\sqrt{{v_{\alpha }}^{2}+{v_{\beta }}^{2},}$$

(7) $$\tan\left(\theta \right)=\frac{v_{\beta }}{v_{\alpha }}.$$

Then, substituting (4) and (5) in (7), the module of the reference vector can be redefined as

$$|V_{ref}|=\sqrt{{\left(V_{m}\cos\left(\omega t\right)\right)}^{2}+{\left(V_{m}\sin\left(\omega t\right)\right)}^{2}},$$

(8) $$|V_{ref}|=V_{m},$$

and finally, from (7), the angle θ can be calculated as follows:

(9) $$\theta =\tan^{-1}\left(\frac{v_{\beta }}{v_{\alpha }}\right).$$

**Figure 3:**
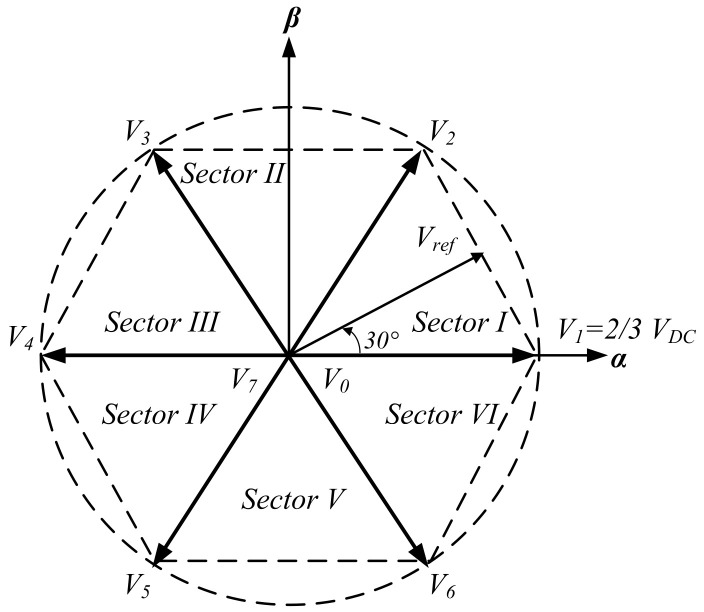
Space vector representation of the DCM-232 states.

From the above analysis, the eight states of the DCM-232 inverter can be represented on the α-β plane, as shown in Figure 3. Considering this representation, it should be noted that this is similar to that of the space vector representation for a 3P-FB inverter; however, in this particular case, the zero vectors imply the decoupling of the DC sources performed by switches $S_{7a}$, $S_{7b}$, $S_{8a}$, and $S_{8b}$.

Considering the polar representation, the eight vectors in the complex plane can be written as

(10) $$V_{n}=\left\{\begin{array}{cc} \frac{2}{3}V_{DC}e^{j\frac{\pi }{3}\left(n-1\right)} & n=1,\ldots ,6\\ 0 & n=0,7 \end{array}\right.$$

For a balanced three-phase system, $V_{ref}$ can be expressed as

(11) $$V_{ref}=Ve^{j\omega t}.$$

In order to synthesize $V_{ref}$, three successive space vectors can be applied along a switching period ($T_{s}=\frac{1}{f_{s}}$). Therefore, the addition of the applied vectors (active and/or null) must satisfy

(12) $$V_{a}t_{a}+V_{b}t_{b}+V_{N}t_{0}=V_{ref}T_{s},$$

Notice that the switching period is the sum of the times of each applied vector:

(13) $$t_{a}+t_{b}+t_{0}=T_{s}.$$

To determine the duty cycles for each applied vector, the complex components ($V_{A}$ and $V_{B}$) in the α-β plane for $V_{ref}$ can be defined as

(14) $$V_{ref}=\left\{\begin{array}{c} V_{A}=\frac{1}{T_{s}}V_{a}t_{a}\\ V_{B}=\frac{1}{T_{s}}V_{b}t_{b}. \end{array}\right.$$

**Figure 4:**
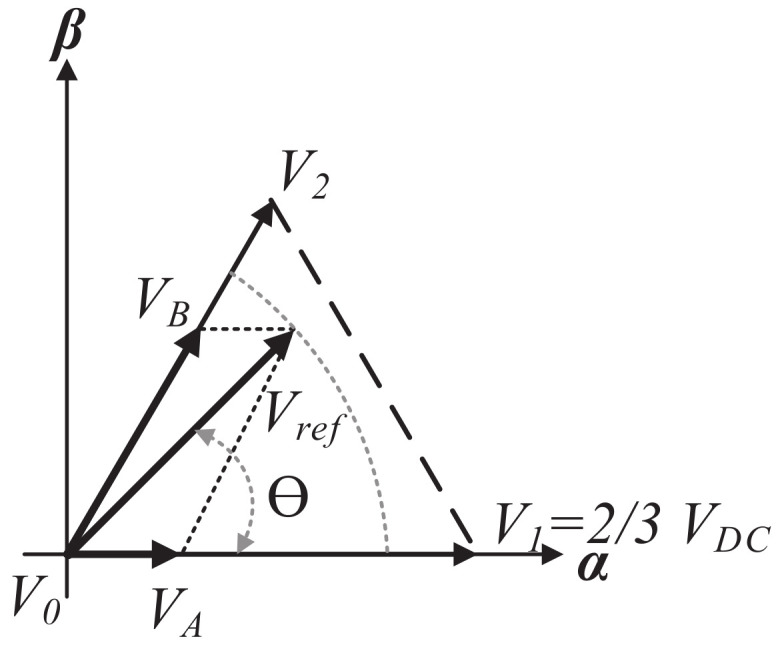
Analysis of $V_{ref}$ at the Sector 1.

The complex components $V_{A}$ and $V_{B}$ defined in (14) can be represented in Sector 1, as shown in Figure 4. As can be observed, $V_{a}=V_{1}$ and $V_{b}=V_{2}$. Therefore, analyzing and performing the projections of $V_{ref}$ over the α and β axis yields

$$|V_{A}|∠0^{\circ },$$

$$|V_{B}|∠60^{\circ },$$

$$|V_{ref}|=V_{m},$$

(15) $$|V_{ref}\left|\cos\left(\theta \right)=\right|V_{A}\left|+\right|V_{B}|\sin\left(60^{\circ }\right),$$

(16) $$|V_{ref}\left|\sin\left(\theta \right)=\right|V_{B}|\sin\left(60^{\circ }\right).$$

Solving for $|V_{B}|$ from (16),

(17) $$|V_{B}|=\frac{|V_{ref}|\sin\left(\theta \right)}{\sin(60^{\circ })};$$

solving for $|V_{A}|$ from (15); and substituting (17) yields

$$|V_{A}\left|=\right|V_{ref}\left|\cos\left(\theta \right)-\right|V_{B}|\sin\left(60^{\circ }\right),$$

$$|V_{A}\left|=\right|V_{ref}|\left(\frac{\sin\left(60^{\circ }\right)\cos\left(\theta \right)-\cos\left(60^{\circ }\right)\sin\left(\theta \right)}{\sin(60^{\circ })}\right).$$

Then, using the following trigonometric identity,

sin(a−b)=sin(a)cos(b)−cos(a)sin(b),

yields

(18) $$|V_{A}|=\frac{|V_{ref}|\sin\left(60^{\circ }-\theta \right)}{\sin(60^{\circ })}.$$

Substituting the components (14) in (17) and (18),

$$\frac{1}{T_{s}}V_{b}t_{b}=\frac{|V_{ref}|\sin\left(\theta \right)}{\sin(60^{\circ })},$$

$$\frac{1}{T_{s}}V_{a}t_{a}=\frac{|V_{ref}|\sin\left(60^{\circ }-\theta \right)}{\sin(60^{\circ })}.$$

Now, solving for $t_{a}$,

$$t_{a}=\frac{|V_{ref}|T_{s}\sin\left(60^{\circ }-\theta \right)}{V_{a}\sin\left(60^{\circ }\right)},$$

and then solving for $t_{b},$

$$t_{b}=\frac{|V_{ref}|T_{s}\sin\left(\theta \right)}{V_{b}\sin\left(60^{\circ }\right)},$$

and since $V_{a}=V_{b}=\frac{2}{3}V_{DC}$ and $\sin\left(60^{\circ }\right)=\frac{\sqrt{3}}{2},$

(19) $$t_{a}=\frac{\sqrt{3}\left|V_{ref}\right|T_{s}\sin\left(60^{\circ }-\theta \right)}{V_{DC}}$$

and

(20) $$t_{b}=\frac{\sqrt{3}\left|V_{ref}\right|T_{s}\sin\left(\theta \right)}{V_{DC}}.$$

Finally, solving for $t_{0}$ from (13) yields

(21) $$t_{0}=T_{s}-t_{a}-t_{b}.$$

**Figure 5:**
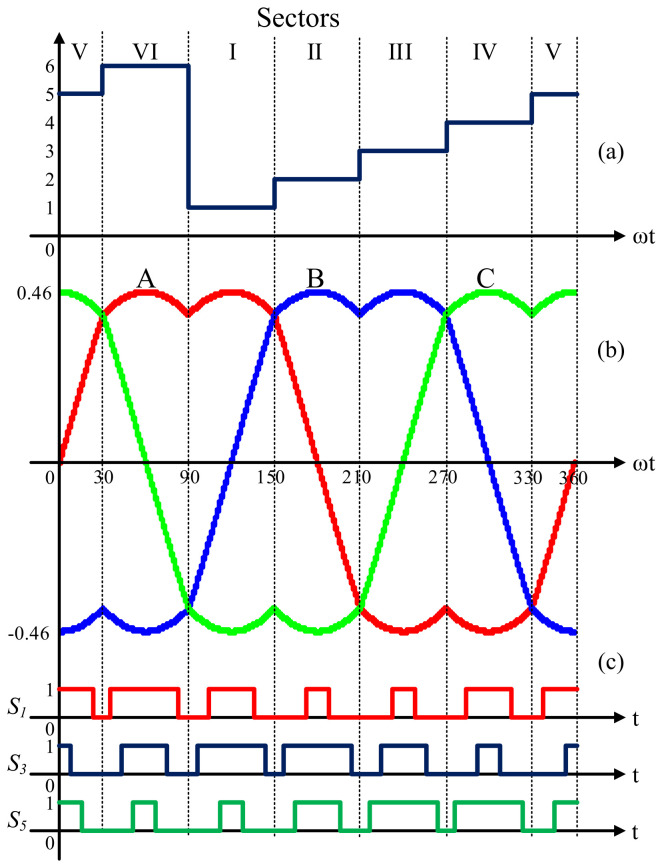
From top to bottom, (**a**) sectors; (**b**) evolution of the switching times $t_{a}$, $t_{b}$, and $t_{0}$; and (**c**) switching sequences for $S_{1}$, $S_{3}$, and $S_{5}$ in a conventional three-phase full-bridge inverter.

Equations (19)–(21) are a general solution for $t_{a}$, $t_{b}$, and $t_{0}$ since the times used for each active or null vector along the grid period are the same. The evolution of the calculated times and PWM switching signals along a grid period is depicted in Figure 5, where *A* is the waveform for the evolution of $t_{a}$, *B* is the waveform for the evolution of $t_{b}$, and *C* is the waveform for the evolution of $t_{0}$.

By using these time calculations and by considering that the main objective of the DCM-232 topology is to reduce the CMC by decoupling the DC sources, a simple way to obtain a constant common mode voltage for different vector sequences is proposed. Considering the active vectors given in Table 1 and the operation states of the DCM-232 inverter as logic states, the following Boolean expressions are obtained:

(22) $$S_{7}=S_{1}\bar{S_{3}S_{5}}+\bar{S_{1}}S_{3}\bar{S_{5}}+\bar{S_{1}S_{3}}S_{5},$$

(23) $$S_{8}=S_{1}S_{3}\bar{S_{5}}+\bar{S_{1}}S_{3}S_{5}+S_{1}\bar{S_{3}}S_{5}.$$

According to (22) and (23), the switches $S_{7}$ and $S_{8}$ are in the active state when the corresponding logic states involved in each equation comply with the logic conditions. Therefore, only when the active vectors appear in the modulation sequences are $S_{7}$ and $S_{8}$ turned on.

**Figure 6:**
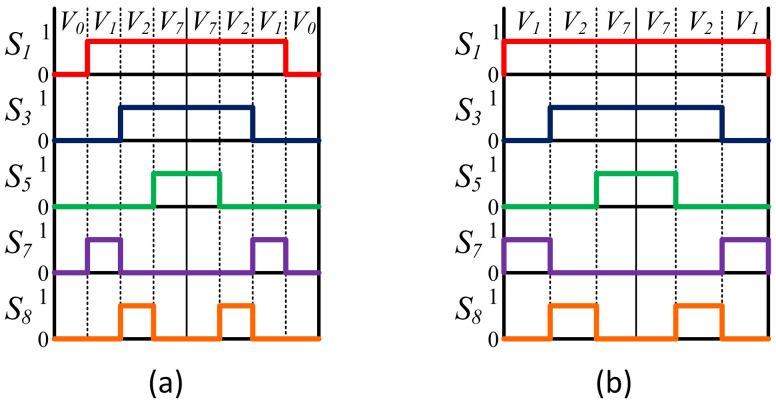
Switching patterns for (**a**) CSSVM and CASVM, and (**b**) DSVMMAX.

Based on the above analysis, three different modulation strategies are proposed in this paper to control the DCM-232 topology using the proposed technique. Note that any vector sequence can be adopted to control the inverter. In this paper, the proposed SVM strategies are based in the conventional SVM for a three-phase full-bridge inverter, named Conventional Symmetric Space Vector Modulation (CSSVM), Conventional Asymmetric Space Vector Modulation (CASVM), and Discontinuous Space Vector Modulation Maximum (DSVMMAX). The switching patterns for these three proposed SVM strategies are depicted in Figure 6. Note that the switching pattern for CSSVM and CASVM strategies is the same, and the main difference is the way in which the times $t_{a}$, $t_{b}$, and $t_{0}$ are computed, as shown below.

## 3. Numerical Results

**Figure 7:**
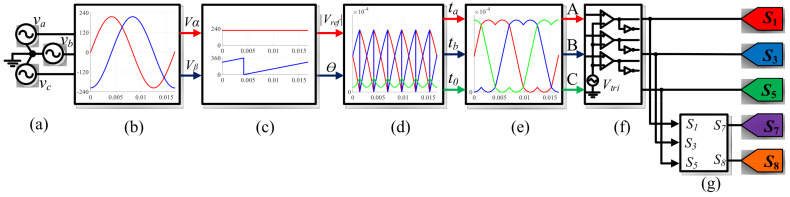
Block diagram of the implemented system in the PSIM software: the block (**a**) represents the three-phase signals; (**b**) is the Clarke transformation; (**c**) is the module and angle of the reference vector; (**d**) is the time vectors calculation; (**e**) reference signals for space vectors; and (**f**,**g**) represent the PWM signal generation.

**Figure 8:**
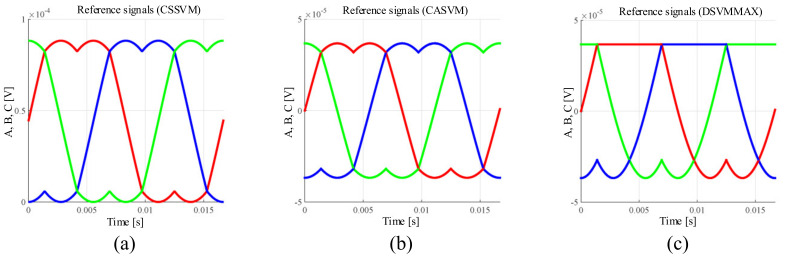
Reference signals for (**a**) CSSVM, (**b**) CASVM, and (**c**) DSVMMAX.

**Figure 9:**
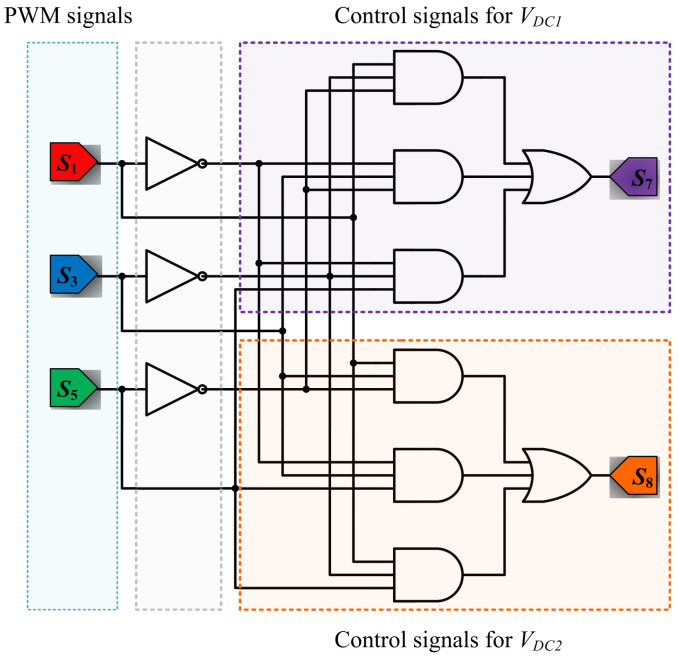
Digital circuit to generate the PWM signals for S7 and S8 switches.

To validate the proposed SVM technique for the three-phase DC-232 inverter, the numerical results are reported using the parameters shown in Table 3. It is important to highlight that the numerical results are obtained in an open loop configuration, since the main objective is to validate the proposed SVM techniques operating with this topology. The general scheme implemented in the PSIM software is shown in Figure 7, where (a) the three-phase signals, (b) the Clark transformation, (c) the module and angle of the reference vector, (d) time vector calculation, (e) the reference signals for the space vectors, and (f and g) PWM signal generation are presented. In the particular case of the block (e), the reference signals are defined in different ways for the three-proposed SVM strategies. In Figure 8, the reference signals for CSSVM, CASVM, and DSVMMAX are depicted. Note that the reference signals for CSSVM have only positive values, CASVM is centered at zero and has positive and negative values, and DSVMMAX is centered at zero but has an unsymmetrical waveform. Note that these reference signals are generated by the addition of the time intervals calculated for each vector along each sector and their magnitude is related to the switching period. Moreover, the block depicted in (f) is dedicated to generate the PWM signals, in particular, the signals for the switches on the DC side are generated using the digital circuit depicted in Figure 9 according to (22) and (23).

**Figure 10:**
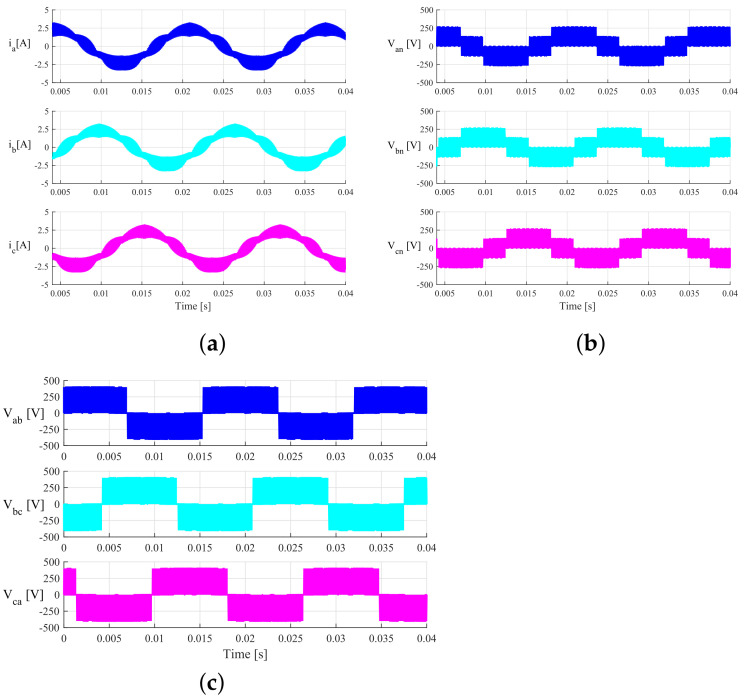
Output currents and voltages of the three-phase DCM-232 inverter under SVM-CSSVM technique. (**a**) Three-phase output currents, (**b**) line-to-neutral voltages, and (**c**) line-to-line voltages.

**Figure 11:**
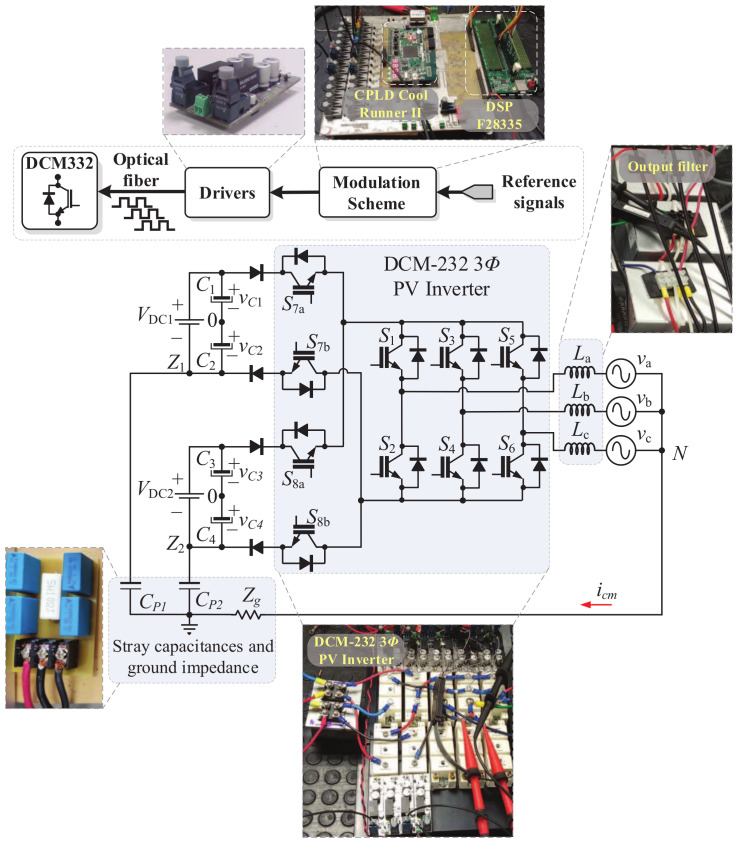
Experimental setup of the DCM-232 inverter.

**Figure 12:**
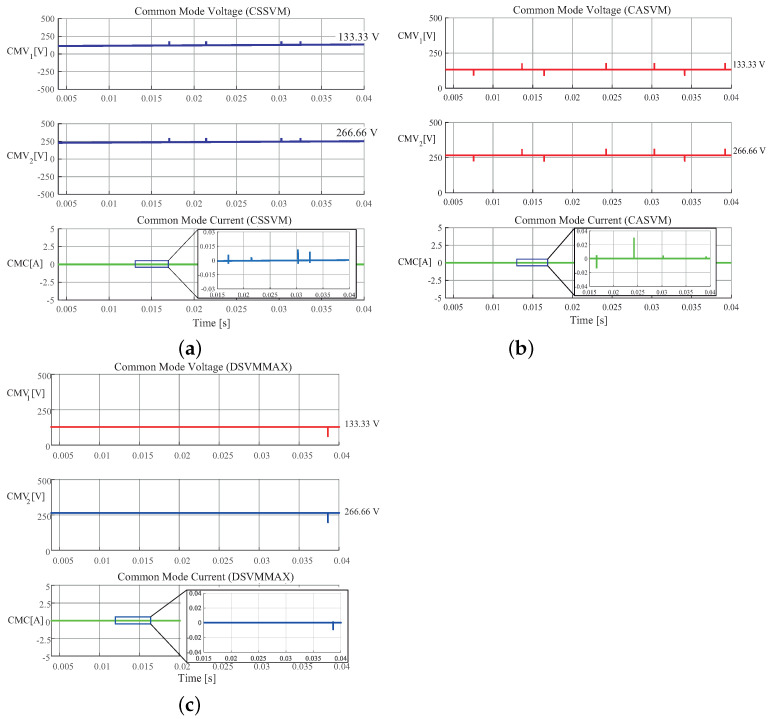
Simulation results of common mode voltage (CMV) and common mode current (CMC) of the three-phase DCM-232 inverter under the (**a**) CSSVM, (**b**) CASVM, and (**c**) DSVMMAX techniques.

The numerical results were obtained for the three SVM techniques; however, the waveforms for the output currents and voltages are very similar in the three cases. Therefore, for brevity, only the waveforms for the CSSVM technique are included. Figure 10 shows the simulation results for the three-phase output currents, line-to-neutral voltages, and line-to-line voltages. As observed, these waveforms are similar to those typical waveforms of a three-phase conventional inverter. It can be observed also that the switching ripple appears at the sinusoidal current waveforms in which peak current is around 2.5 A. It is important to note that, under these conditions, the ripple magnitude is large and the measured THDi is around 16%, which is not allowed by the international standard, for instance, IEEE 519-2014 (considering a low grid voltage). It should be also noted that, in this case, a first-order low-pass filter is used at the output of the inverter (see Figure 11), so this can be improved by implementing a third-order filter. It is possible to increase the switching frequency or the rated power to improve the THDi as well; however, in this case, these parameters are limited by the experimental setup. On the other hand, the common mode voltage and current are also obtained by simulations; in this case, the three sets of results are presented in Figure 12. As it can be observed, the results show that these two parameters are similar for the three proposed cases. In the case of the CMV, the magnitude is predominantly constant, and in the case of the CMC, the maximum value is around 40 mA, which is well below the limits imposed by the DIN VDE 0126-1-1 international standard, which is up to 300 mA (RMS). It should be noted that the numerical simulations were performed considering a balanced DC-bus, that is $V_{DC1}=V_{DC2}$. However, in a transformerless PV application, when the irradiance changes along a day, the voltage at the maximum power point also changes. This variation, which is typically around 10% to 20%, produces a DC component at the output of the DCM-232 inverter. Under these conditions, the inverter is still capable of operating but with a DC component which is not allowed. To solve this problem, a balance technique should be implemented, and this can be solved either by implementing a balance control loop or by modifying the modulation strategy; however, this issue is out of the scope of this paper.

**Figure 13:**
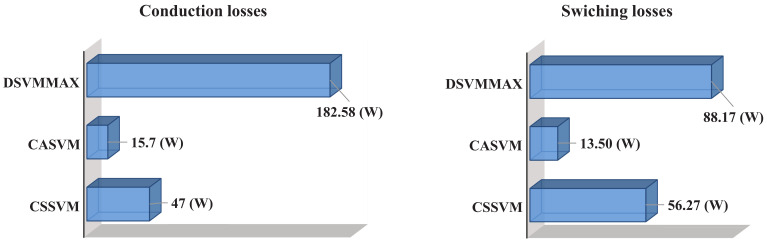
Switching and conduction losses of the DCM-232 inverter under the three SVM strategies.

To better compare the three SVM strategies under study, an efficiency analysis was performed. For this purpose, the IGBT model was loaded into the Thermal Module of the PSIM software and the total losses of the DCM-232 converter were calculated. The model loaded in the software considers the parameters provided by the manufacturer; then, the behavior of the power losses is expected to be close to the real behavior. The results of the switching and conduction losses are shown in Figure 13. As can be observed, the sum of the switching and conduction power losses is greater for the DSVMMAX with respect to the other techniques, while the CASVM presents the lowest total power losses. Therefore, the CASVM should be expected to present the highest efficiency. To validate this parameter, the efficiency of the system was also measured and the results are presented in Table 4, where, as expected, the CASVM technique presents the best efficiency.

### Considerations for a Practical Implementation

**Figure 14:**
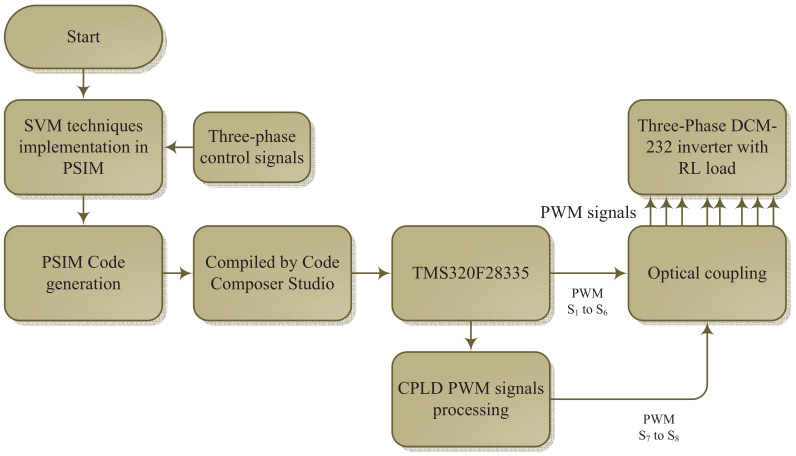
Flowchart for the experimental implementation.

The DCM-232 three-phase inverter was implemented as a laboratory prototype to validate the proposed SVM strategies. A flow chart of the implementation process is shown in Figure 14. The algorithms for the SVM strategies were implemented using a Digital Signal Processor (DSP) TMS320F28335 device together with the PSIM software. Additionally, the digital functions for the PWM signals were implemented in a complex programmable logic device (CPLD) CoolRunner-II according with Figure 14. The power module SKM50GB12T4 was used to implement the DCM-232 three-phase inverter, and the diodes $D_{1}$ to $D_{4}$ were implemented using the power module 200RD4TVL. The electrical parameters are in accordance with the parameters used for the simulation test, as listed in Table 3. A simplified block diagram of the experimental setup is depicted in Figure 11. The ground path was implemented by connecting the neutral point of the RL load to the available terminal of the parasitic capacitors $C_{p1},C_{p2},C_{p3}$, and $C_{p4}$ through a resistance with a value equal to 22 Ω.

## 4. Experimental Validation

**Figure 15:**
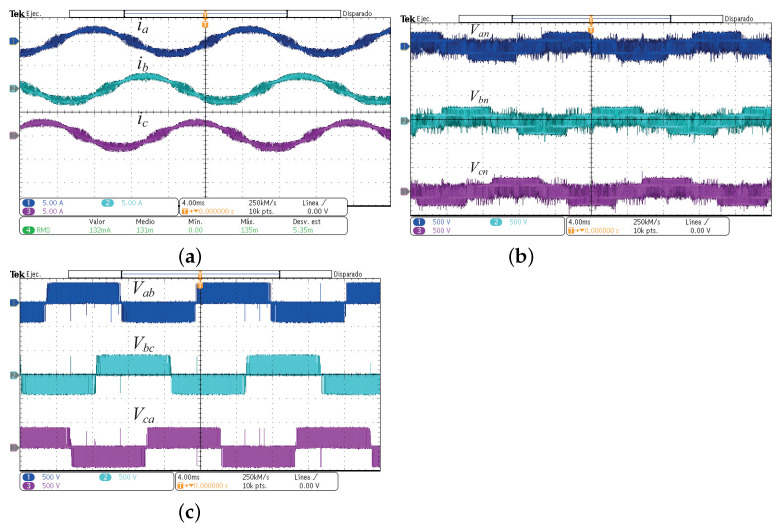
Experimental output currents and voltages of the three-phase DCM-232 inverter under the SVM-CSSVM technique. (**a**) Output currents, (**b**) line-to-neutral voltages, and (**c**) line-to-line voltages.

**Figure 16:**
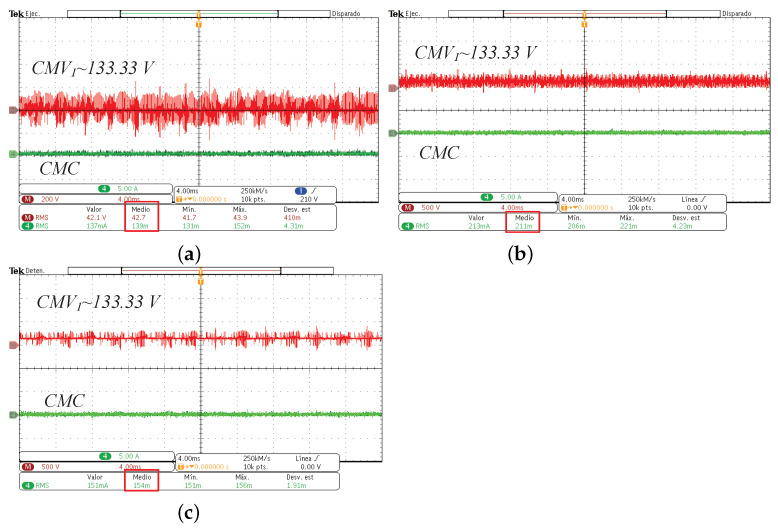
Experimental common mode voltage ($CMV_{1}$) and common mode current (CMC) of the three-phase DCM-232 inverter under the (**a**) CSSVM, (**b**) CASVM, and (**c**) DSVMMAX techniques.

The experimental implementation was performed considering the parameters and conditions described in Section 3 and in Table 3. In Figure 15, the output currents in (a), the line-to-neutral voltages in (b), and the line-to-line voltages in (c) for the CSSVM strategy are presented. As can be observed, the waveforms are similar to those obtained for the simulation results. Namely, the output current for the three phases is sinusoidal plus the switching ripple, the voltage between each phase and the neutral connection has the typical five levels, and the voltages between lines are also the typical of a full-bridge three-phase system. Since these waveforms are close to the waveforms of the other two proposed SVPWM strategies, only the results for the CSSVM are included. In Figure 16, the results obtained for the common mode behavior for (a) CSSVM, (b) CASVM, and (c) DSVMMAX at the DC bus 1 and the common mode current are shown. Notice that, in all cases, the CMC has a value below 300 mA, which is established by the international norm DIN VDE 0126-1-1 as the maximum allowable limit. Note that the $CMV_{1}$ waveform contains a noise component, which is due to the oscilloscope internal calculations and the effect of parasitic components during the switching process. Moreover, the CMV regarding the second DC bus $CMV_{2}$ has also been obtained. The average value of this parameter is close to 266.66V, which is the value obtained by means of simulations; however, and considering that the waveform is similar to the signal presented for the $CMV_{1}$, the last is not included in the paper.

In order to better compare the results obtained by the implementation of the proposed SVM algorithms regarding CMC, the measures are summarized in Table 5. As can be noted, the RMS value for the CMC for the three proposed cases is similar and complies with the international standard mentioned before. Moreover, the results regarding CMC were compared with the conventional full-bridge three-phase inverter (3PFB-VSI) under SVM, and it can be noted that the CMC has a larger magnitude regarding the proposed modulation topology and SVPWM algorithms.

## 5. Conclusions

In this paper, three hybrid modulation strategies were proposed. The proposed modulation strategies combine the time calculations of the space vector modulation technique and the comparison of the reference signals with a carrier triangular waveform, as does the sinusoidal pulse width modulation strategies. The results show that the SVM algorithms for the DCM-232 can be implemented using this combined method, which is convenient for practical implementation in a digital platform. Moreover, an evaluation of the efficiency was performed, and the results show that the modulation sequence has an important effect on this parameter. This analysis demonstrates that the CASVM technique has the highest efficiency (95.85%) among the proposed modulations algorithms, although all of the proposed methods present an efficiency above 85.5%. Finally, it can be also concluded that any of the proposed techniques is capable of reducing the magnitude of the common mode current (i.e., up to 140 ${\mathrm{mA}}_{\mathrm{RMS}}$ for the CSSVM technique), which is a very interesting feature for transformerless photovoltaic applications since the power inverter operation complies with the international standards. 

## Figures and Tables

**Table 1:** Switching configurations of the three-phase DCM-232 transformerless inverter.

State (Vector)	$S_{1}$	$S_{3}$	$S_{5}$	$S_{7a}$/$S_{7b}$	$S_{8a}$/$S_{8b}$	$v_{\mathrm{aN}}$ (V)	$v_{\mathrm{bN}}$ (V)	$v_{\mathrm{cN}}$ (V)
** $V_{0}$ **	0	0	0	0	0	0	0	0
** $V_{1}$ **	1	0	0	1	0	$\frac{2V_{DC1}}{3}$	$\frac{-V_{DC1}}{3}$	$\frac{-V_{DC1}}{3}$
$V_{2}$	1	1	0	0	1	$\frac{V_{DC2}}{3}$	$\frac{V_{DC2}}{3}$	$\frac{-2V_{DC2}}{3}$
** $V_{3}$ **	0	1	0	1	0	$\frac{-V_{DC1}}{3}$	$\frac{2V_{DC1}}{3}$	$\frac{-V_{DC1}}{3}$
$V_{4}$	0	1	1	0	1	$\frac{-2V_{DC2}}{3}$	$\frac{V_{DC2}}{3}$	$\frac{V_{DC2}}{3}$
$V_{5}$	0	0	1	1	0	$\frac{-V_{DC1}}{3}$	$\frac{-V_{DC1}}{3}$	$\frac{2V_{DC1}}{3}$
$V_{6}$	1	0	1	0	1	$\frac{V_{DC2}}{3}$	$\frac{-2V_{DC2}}{3}$	$\frac{V_{DC2}}{3}$
** $V_{7}$ **	1	1	1	0	0	0	0	0

**Table 2:** Switching configurations of the three-phase DCM-232 transformerless inverter.

State (Vector)	$V_{\mathrm{CMV}1}$	$V_{\mathrm{CMV}2}$
$V_{0}$ to $V_{7}$	$\frac{V_{DC}}{3}$	$\frac{2V_{DC}}{3}$

**Table 3:** Simulation and experimental parameters.

Parameter	Value
$V_{DC1},V_{DC2}$	400 V
$f_{s}$	10 and 12 kHz
$t_{d}$	1 µs
$L_{a},L_{b},L_{c}$	2 mH
$R_{a},R_{b},R_{c}$	71.43 Ω
$Z_{g}$	22 Ω
$C_{1},C_{2},C_{3},C_{4}$	2200 µF
$C_{p1},C_{p2},C_{p3},C_{p4}$	160 nF
$M_{i}$	0.8

**Table 4:** Efficiency of the DCM-232.

SVM Strategy	Efficiency (%)
CSSVM	85.87
CASVM	95.85
DSVMMAX	85.50

**Table 5:** CMC magnitudes of the DCM-232 under the proposed SVPWM strategies.

SVM Strategy	CMC (${\mathrm{mA}}_{\mathrm{RMS}}$)
CSSVM	140
CASVM	145
DSVMMAX	156
3PFB-VSI	1630

## Abbreviations

The following abbreviations are used in this paper:

PWM	Pulse Width Modulation
DC	Direct Current
DC-AC	Direct Current–Alternating Current
PV	Photovoltaic
LKC	Leakage Currents
3P-CMI	Three-Phase Cascade Multilevel Inverter
CMV	Common-Mode Voltage
CMC	Common-Mode Current
THD	Total Harmonic Distortion
RMS	Root Mean Square
NPC	Neutral Point Clampled
3P-FB	Six-Switch Three-Phase Inverter
NSPWM	Near-State Pulse Width Modulation
3P-FBSC	Six-Switch Three-Phase Inverter with Split Capacitor
3P-NPC	Three-Phase Neutral Point Clamped Inverter
SVM	Space Vector Modulation
IGBT	Isolated Gate Bipolar Transistor
MOSFET	Metal-Oxide Semiconductor Field-Effect Transistor
HERIC	Highly Efficient and Reliable Inverter Concept
SVPWM	Space Vector Pulse Width Modulation
CPLD	Complex Programmable Logic Device
CMM	Common-Mode Model
CSSVM	Conventional Symmetric Space Vector Modulation
CASVM	Conventional Asymmetric Space Vector Modulation
DSVMMAX	Discontinuous Space Vector Modulation Maximum
DSP	Digital Signal Processor
